# Introducing *Plant Direct*

**DOI:** 10.1002/pld3.1

**Published:** 2017-06-28

**Authors:** Ivan Baxter

As the Editor‐in‐Chief, I am excited to introduce *Plant Direct,* a new journal from Wiley and the societies behind *Plant Physiology*,* The Plant Journal,* and *The Plant Cell*. While there is a crowded landscape of journals to choose from, we believe that *Plant Direct* fills an unserved role for the plant community. We seek to be the sound science plant journal for the community of ASPB members, SEB members, and the authors who publish in our society journals. By “sound science,” we mean that instead of trying to determine the novelty or impact of a paper, we will judge manuscripts based on whether the experiments and analysis are performed to an acceptable technical standard and described in adequate detail in standard English, how well the conclusions are supported by the data, and whether the manuscript and contents meet all ethical and research integrity standards (as the person who will have to deal with it if it occurs, can we please not violate the last one, pretty please?).

*Plant Direct* is a society journal, and we think the service to community part of our mission statement is incredibly important. Publishing has changed dramatically over the past two decades, and many of the services that journals have provided are no longer essential. The single most important service that journals provide now is managing the peer review process. But the effort of peer review at a sound science journal is made entirely by the community, active scientists are both the editors and reviewers (helped out by the great management staff at Wiley). When the community is doing the work, the community should reap the benefits. Our societies support our community, and the two sponsoring societies, ASPB and SEB, will be able to use the revenues from this journal to support their important work in education, outreach, networking, and the advancement of plant science.

Starting a new journal also allowed us to look at the way we run our journals with a fresh eye. The past 20 years have seen a wide variety of innovations in scientific publishing, including journals moving online and the proliferation of open‐access journals. Nonetheless, for many researchers the publishing process continues to be slow, frustrating, and wasteful of authors’ and reviewers’ time. Manuscripts pass through multiple stages of review and revision, sometimes spanning multiple journals, each using new reviewers. Although this process improves many papers, it also greatly delays when the science becomes available to the scientific community. There are three features of *Plant Direct* that will improve the speed, efficiency, and openness of the publication process:

1. Strong promotion of preprint posting at submission.
2. Referred reviews from *Plant Physiology*,* The Plant Cell,* and *The Plant Journal—*that is, manuscripts are easily transferred for consideration along with any accompanying reviewer reports.
3. Publication of anonymous reviewer reports upon acceptance.

These features will speed scientific communication in two ways. The preprint is visible to the scientific community immediately, although readers must judge for themselves the work's technical merit. This visibility has major benefits for authors, especially junior scientists applying for fellowships, grants, and jobs, by showing their great science to the world. It also benefits readers who are not in the author's close‐knit network and thus may not be able to put the author's hard work to use to move their field forward until it is published as a peer‐reviewed article. We think that preprints are such a large benefit to the community that we will give you a discount on your publication charges if your manuscript is posted on a preprint server at the time of submission to *Plant Direct*.

The other features of *Plant Direct* speed the publication process by reducing or eliminating steps that contribute to delays. Allowing transfer of manuscripts from referring journals, or from the bioRxiv preprint server, will save authors the hassle of entering manuscript metadata multiple times. Referred reviews from participating journals allow the editor to benefit from previous reviews and may remove altogether the need to send the manuscript out for review, thereby reducing reviewer workload. The publication of reviewer comments will make the process more open and help to ensure the integrity and transparency of the review process.

We are also going to be working with our author, reviewer, and editor community to identify and implement new ways to reduce the bottlenecks in the publication process. We will host a series of discussions in spring/summer 2017, both online (at Plantae.org) and at scientific meetings, to brainstorm new ideas and gauge the support for these ideas and ideas already in circulation. Please let us know your thoughts on this idea as well as how we can make the publishing process better. We are also interested in ideas on how to incorporate mentorship and training of postdoctorals and graduate students into the publishing process.

Let me conclude by asking you, the plant community, to join us as we launch this community journal. Please send us your manuscripts, review our manuscripts, and tell us how we can make the journal and the community better. We are excited to get this started and hope that you will be too.

